# Effective Resistance to Four Fungal Foliar Diseases in Samples of Wild *Triticum* L. Species from the VIR (N.I. Vavilov All-Russian Institute of Plant Genetic Resources) Collection: View from Vavilov’s Concepts of Plant Immunity

**DOI:** 10.3390/plants11243467

**Published:** 2022-12-10

**Authors:** Lev G. Tyryshkin, Natalia S. Lysenko, Maria A. Kolesova

**Affiliations:** Federal Research Center N. I. Vavilov All-Russian Institute of Plant Genetic Resources (VIR), Bolshaya Morskaya Str. 42–44, 190000 Saint Petersburg, Russia

**Keywords:** wild *Triticum* L. species, *T. boeoticum*, *T. urartu*, *T. araraticum*, *T. dicoccoides*, SNB, *Pt*, HLB, *Bgt*, seedling resistance, adult resistance, resistance genes

## Abstract

To identify new sources of effective resistance to four foliar diseases of wheat, 173 accessions of four wheat species, *Triticum boeoticum*, *T. urartu*, *T. araraticum*, and *T. dicoccoides*, from the VIR collection were tested at the juvenile and adult growth stages for resistance to leaf rust (*Pt = Puccinia triticina*), powdery mildew (*Bgt = *Blumeria graminis tritici**), Septoria nodorum blotch (SNB), and dark-brown leaf spot blotch (HLB = Helminthospjrium leaf blotch). The accessions included new additions to the collection, some old samples that had never been tested before, as well as earlier tested samples noted for high levels of juvenile resistance to some fungal diseases. Natural populations of *Puccinia triticina* and *Blumeria graminis* f. sp. *tritici*, mixture of *Parastagonospora nodorum* and *Bipolaris sorokiniana* isolates were used to inoculate and to evaluate resistance to *Pt*, *Bgt*, SNB, and HLB, respectively. Two samples of *T. boeoticum*, three of *T. urartu*, and one of *T. araraticum* were resistant to leaf rust at both tested stages. Further tests (phytopathological and molecular analyses) excluded *Lr9, Lr19, Lr24, Lr41,* or *Lr47* as single genes controlling resistance; hence, these accessions likely carry new effective leaf rust resistance genes. High level of *Bgt* resistance was identified in three entries of *T. boeoticum*, one of *T. araraticum*, and eleven of *T. dicoccoides*. All tested accessions were susceptible to HLB and SNB at both tested stages. Accessions identified as resistant are valuable plant material for introgressive hybridization in bread and durum wheat breeding. The results are discussed in the context of N.I. Vavilov’s concept of crop origin and diversity, and the laws of plant natural immunity to infectious diseases.

## 1. Introduction

Bread wheat (*Triticum aestivum* L.) is one of the most important cereal crops in the Russian Federation (RF) as well as all over the world. It is the main product of grain export for the RF. The yield of the crop and its quality are significantly reduced because of foliar fungal diseases. Leaf rust (*Pt*) (causal agent—*Puccinia triticina* Erikss.), *Septoria nodorum* blotch (SNB) (*Parastagonospora nodorum* (Berk.) Quaedvlieg, Verkley, and Crous syn. *Stagonospora nodorum* (Berk.) Castell. et Germano), dark-brown leaf spot blotch (indicating Helminthosporium leaf blotch (HLB)) (*Bipolaris sorokiniana* (Sacc.) Shoemaker, teleomorph *Cochliobolus sativus* (Ito et Curib.), and powdery mildew (*Bgt*) (*Blumeria graminis* (DC.) E.O. Speer f. sp. *tritici* Em. Marchal syn. *Erysiphe graminis* f. sp. *tritici*) are devastating diseases of common wheat in many of the crop-growing regions. Under severe epiphytotic conditions, yield losses from leaf rust development have been estimated to occur in up to 14 percent of the total wheat yield for the Great Plains of North America [1], and in more than 40% in Russia [2]. In the Southern Cone of South America *Pt* can cause grain yield losses higher than 50% during severe epidemics if fungicides are not applied [3]. Leaf rust infections at earlier stages of wheat growth may also cause yield losses higher than 50% [4]. Losses up to 50% due to SNB have been reported [5]. HLB can result in yield losses up to 100% in susceptible varieties under favorable conditions [6]. Powdery mildew causes yield losses as high as 48% [7]. Yield losses from *Bgt* under severe epiphytotic conditions in Russia can reach 50% or more [8]. In addition to yield losses, the diseases decrease the qualities of the wheat grains [5,9,10,11].

The most effective and environmentally friendly method to avoid economically significant losses of wheat yield from these, as well as other diseases, has been well proven to be the cultivation of resistant varieties. To develop such varieties, the sources and donors of effective genes and genetic systems for the resistance are needed.

The collection of the All-Russian Institute of Plant Genetic Resources (VIR, Saint Petersburg, Russia), the basis of which was established by N.I. Vavilov, represents a very valuable plant material for searching sources and donors and for breeding important traits, including resistance to diseases [12,13]. However, our previous work with bread wheat from this collection showed very narrow genetic diversity for effective resistance to modern populations of leaf rust, HLB, SNB, and powdery mildew pathogens from the Russian geographic habitat [14,15].

All samples from the Vavilov’ collection with effective seedling resistance to leaf rust are protected with genes *Lr9*, *Lr19*, *Lr24*, and *Lr41* [14,15,16]. These genes have been highly effective in many regions of the world, but now virulence to genes *Lr9*, *Lr19*, and *Lr24* has been shown in Russia [17,18,19,20]. Pathotypes virulent to *Lr9* have been found in the United States [21], Canada [22], Mexico [23], the Czech Republic [24], and in Egypt [25]. Pathotypes virulent to *Lr19* have been found in Mexico [26], India [27], the Czech Republic [24], and in Egypt [25]. Virulence for *Lr24* has occurred in the United States, Argentina, Brazil [21], South Africa [21,28] and other southern Africa countries [29], in south Australia [30], and in Canada [22]. Clones virulent to *Lr41* have been found in the United States [31]. Evidently wide commercial growing of varieties with these genes will result in rapid accumulation of virulent pathogen genotypes and, hence, loss of resistance.

Not a single bread wheat sample from the VIR collection possessed high resistance to SNB and HLB at different stages of plant growth [14,15,16].

The low genetic diversity for effective resistance to diseases in *T. aestivum* presents the urgent task of expanding it through various approaches, including interspecific hybridization with related species in the *Triticum* genus. Study of seedling resistance to the diseases in cultivated species of the genus did not reveal significant genetic diversity for effective resistance [14,15,16,32]. Thus, special attention should be paid to wild *Triticum* L. wheat species [12], which are represented by: wild einkorn wheat *T. boeoticum* Boiss., red wild einkorn *T. urartu* Thum. ex Gandil., Armenian wild emmer *T. araraticum* Jakubz., and wild emmer *T. dicoccoides* (Koern. ex Aschers. et Graebn.) Schweinf. Some samples of such species from the VIR collection were earlier screened for resistance to diseases [12,14,15,16,33,34,35,36,37,38]. For example, in the Vavilov study all collection samples of *T. boeoticum*, *T. urartu*, and those of *T. dicoccoides* from Syria were resistant to leaf rust, while samples of *T. dicoccoides* from Palestine were differentiated for the resistance [12]. Some samples of *T. dicoccoides* have been described as resistant to leaf rust and 86% of the samples as resistant to powdery mildew, almost all entries of *T. boeoticum* were characterized by their immunity to these diseases, and *T. araraticum* has been described as highly resistant to *Bgt* [37].

In 1990, in a study of juvenile resistance to SNB, Yamaleev et al. [34] described *T. boeoticum* and *T. urartu* to offer the highest resistance to *S. nodorum* among the *Triticum* species. However, in our previous studies, such a result was not confirmed; all entries of these species were highly susceptible to the disease [14,15,16,32]. Mikhailova et al. [36] revealed 28, 50, 44, and 47% of VIR samples to be resistant and moderately resistant to dark-brown leaf spot blotch in *T. dicoccoides, T. araraticum, T. urartu,* and *T. boeoticum*, respectively. However, our work [14,15,16,32] has indicated that the predominant number of these species’ samples were highly susceptible to the disease. Samples resistant at the juvenile stage to leaf rust were found among all wild wheats from the VIR collection [35]; however, later genotypes resistant to the disease were only found in *T. boeoticum* [14,32]. As for *Bgt*, resistant entries with high frequencies were identified in *T. boeoticum*; all samples of *T. araraticum* were resistant to the disease and among 13 *T. urartu* samples, two were highly resistant [35].

There are several reasons to study resistance to these diseases in wild wheat samples from the VIR collection. There are evident contradictions in earlier obtained results for frequencies of samples resistant to certain disease. Several samples were introduced into the Vavilov’ collection quite long ago, and newer entries introduced into the collection during the last 10 years had never been studied for resistance to the diseases. Additionally, possible changes in virulence and aggressiveness of the causal agents can lead to current susceptibility of wild wheat samples that had earlier been described as resistant.

The purpose of this research was to study effective resistance to four diseases in wild *Triticum* L. species from the VIR collection and to identify entries with high level of the trait expression.

## 2. Materials and Methods

### 2.1. Plant Material

The plant material included 173 accessions of four wild wheat species (two diploids and two tetraploids) from the Vavilov’ collection (Table 1). In this study, classification of *Triticum* genus adopted in Russia [37,38] was used.

The accessions included 138 new additions to the collection, six old samples that had never been tested before, as well as 29 earlier tested samples noted for high level of juvenile resistance to some fungal diseases [32,35,36,37]. 

All the samples were evaluated for resistance to *Pt*, SNB and HLB. Ninety-seven of these, including 15 samples of *T. boeoticum*, 22 of *T. urartu*, 10 of *T. araraticum*, and 50 of *T. dicoccoides*, were tested for resistance to *Bgt*.

### 2.2. Pathogen Material

Phytopathogens were sampled in the fields of Pushkin and Pavlovsk laboratories of VIR (Russia, Northwest Region) in 2020–2022. 

The leaf rust population was drawn from the inoculums from leaves of several susceptible wheat varieties. Under laboratory conditions, the population was maintained and multiplied on seedlings of susceptible cv. Leningradka in a light chamber (2500 lux, temperature–20–22 °C, 24 h daylight). Under these conditions, the population was virulent / avirulent to seedlings of near-isogenic lines of wheat cv. Thatcher and samples of wheat lines with resistance genes *Lr1*, *Lr2а*, *Lr2с*, *Lr3bg*, *Lr10*, *Lr11*, *Lr12*, *Lr13*, *Lr14а*, *Lr14b*, *Lr15*, *Lr16*, *Lr17*, *Lr18*, *Lr20*, *Lr21*, *Lr22а*, *Lr22b*, *Lr23*, *Lr25*, *Lr26*, *Lr28*, *Lr29*, *Lr27+31*, *Lr32*, *Lr33*, *Lr34*, *Lr35*, *Lr36, Lr37, Lr38*, *Lr43*, *Lr44*, *Lr45*, *Lr46*, *Lr48*, *Lr49*, *Lr51*, *Lr52, Lr57*, *Lr60*, *Lr63*, *Lr64*, *Lr67* / *Lr9*, *Lr19*, *Lr24*, *Lr39* (=*Lr41*), and *Lr47*. 

*B. sorokiniana* was isolated from the diseased wheat and barley leaves in Petri dishes on a semi-selective medium [14,39]. The dishes were incubated at 22–25 °С in the darkness; abundant spores’ production was observed within 10–14 days. Inoculum multiplication was done on CZLU medium in Petri dishes [14,39].

Conidia from the medium surface were transferred into water using scalpel; suspension of spores of five fungus isolates in approximately equal quantities were filtered through a double layer of cheesecloth.

*S. nodorum* was isolated from diseased wheat leaves on Potato Glucose Agar for microbiology in Petri dishes and multiplied on pearl barley in flasks under UV light as earlier described [39]. For plant infections, water suspension consisting of a mixture of six *S. nodorum* isolates was prepared by combining equal numbers of pearl grains with each isolate. Grains were placed into water for 15 min.

Inoculum for powdery mildew was sampled as diseased leaves from susceptible wheat accessions in 2021 and 2022 at Pushkin and Pavlovsk laboratories of VIR. The populations of *Bgt* were used to inoculate seedlings of wheat near-isogenic lines and samples with genes for the resistance *Pm1a*, *Pm2*, *Pm3a*, *Pm3b*, *Pm3c*, *Pm3d*, *Pm4a*, *Pm4b*, *Pm4d*, *Pm5a*, *Pm6*, *Pm7*, *Pm8*, *Pm9*, *Pm10*, *Pm11*, *Pm12*, *Pm16*, *Pm17*, *Pm19*, *Pm28*, *PmKu*, and *PmSp*. All of these were heavily infected with the pathogen populations, so no one particular gene available in this study was effective.

### 2.3. Seedling Resistance Screening

In order to study juvenile resistance to the diseases, 15–20 seeds of each sample were placed on cotton wool rolls wetted with water. Ten to fifteen days later, seedlings at the stage of 1–2 leaves were placed horizontally into cuvettes and sprayed with water suspensions of the pathogens’ spores using a hand atomizer (the concentration of *P. triticina* uredospores was 3 × 10^4^ spores ml^−1^, of *B. sorokiniana* conidia was 4 × 10^4^ spores ml^−1^ and of *S. nodorum* spores was 5 × 10^6^ conidia ml^−1^).

After inoculation, the cuvettes were wrapped with polyethylene, covered with glasses, and placed in the darkness. After 24 h, plants were transferred into the light chamber (see above). For the leaf rust experiments, polyethylene film and glass were removed, and seedlings were returned to a vertical position. When studying SNB and HLB resistance, the cuvettes with seedlings remained wrapped until disease scorings. When studying resistance to *Bgt,* vertical seedlings were powdered with spores of *B. graminis* from field collected leaves; infected plants were sprayed with water and were not covered. The types of reaction to *P. triticina* and *B. graminis* infections were scored on the 12th day after inoculation according to generally accepted scales [40,41] with modifications, where 0—no symptoms; 0;—necrotic spots without pustules formation; 1—very small pustules surrounded by necrosis; 2—medium-sized pustules surrounded by necrosis or chlorosis; 3—large pustules without necrosis; s.p.—single pustules of susceptible type without necrosis; and X—pustules of different types on one leaf. Samples with types 0, 0;, and 1 were classified as highly resistant, 2, s.p. and Х–moderately resistant, and 3—susceptible.

Disease ratings of SNB and HLB were scored on the 7th day after inoculation on a scale, where 0—no symptoms of disease; 1, 2, 3, 4—10, 20, 30, 40% of leaf surface affected; 5—50% and more of leaf surface affected, and 6—the death of the leaves. Samples with the disease scores 0, 1 and 2 after inoculation were considered highly resistant, 3 and 4—possessing moderate level of the resistance, and scores of 5 and 6 were classified as susceptible [39]. 

All samples which showed any level of resistance to certain diseases were reevaluated for the trait in 3–4 additional independent experiments. For all diseases, seedlings of wheat cv. Leningradka were used as susceptible controls and were sown after each five experimental samples.

In order to determine the presence of effective leaf rust resistance genes in those samples, which have been resistant to the population of leaf rust, leaf segments of seedlings 0.8–1.0 cm were placed on cotton wool wetted with solution of KCl (0.48 g L^−1^) + NaH_2_PO_4_ × 2H_2_O (0.66 g L^−1^) + maleic acid hydrazide (10 mg L^−1^) [42]. Leaf segments were sprayed with water suspensions of uredospores of single *P. triticina* clones, which were virulent to seedlings of near-isogenic lines of wheat cv. Thatcher and also to samples of bread wheat lines with resistance genes *Lr9, Lr19, Lr24, Lr41,* and *Lr47* under these conditions. For each gene we used five virulent monopustule isolates that had been earlier independently isolated from complex population [42]. Infections with each clone were undertaken in separate experiments without mixing clones. The wild wheat samples, which showed a high type of reaction to inoculation with at least one clone could be considered as possibly protected with that single particular gene [14]. 

Molecular markers, tightly linked to effective genes for seedling and adult plant leaf rust resistance *Lr9*, *Lr19*, *Lr24*, *Lr41*, and *Lr47* in bread wheat (Table 2) under conditions of the NW of the RF were used to identify the corresponding genes in six resistant samples of *T. boeoticum, T. urartu,* and *T. araraticum.* All these genes were transferred into bread wheat genome from species that differed from wild *Triticum* (*Aegilops umbellulata*, *Thinopirum ponticum*, *Th. ponticum*, *Ae. tauschii,* and *Ae. speltoides*, respectively) [43]. It is notable that several alien genes for resistance from some species were identified with the use of molecular markers in other wheat relatives or wheat lines with genetic material from those relatives [44,45,46], possibly due to the presence of orthologous genes in different genomes. 

Total genomic DNA was isolated from 10-day-old seedlings (five plants) with a micro method proposed by Edwards et al. [47] with modifications by Dorokhov and Klocke [48]. A polymerase chain reaction (PCR) was performed in 25 μL reaction volume containing: 2 μL 50 ng/μL of DNA, 2.5 μL 10 × PCR buffer, 1 μL (2.5 mM) dNTPs, 12.5 pmol of each primer, 16 μL MQ H_2_O, and 0.5 μL (2 U/μ) *Taq* DNA Polymerase. Nucleotide sequences of primers listed in Table 2. The PCR was performed in MiniAmp™ Plus Thermal Cycler (Thermo Fisher Scientific, USA) according to original protocols [31,49,50,51,52]. PCR products were separated in 1.5% agarose gel in 1 × TBE buffer, stained in a solution of ethidium bromide (0.5 mg L^−1^) and visualized under UV light. GeneRuler™ 100 bp Plus DNA. Ladder (Fermentas) was used to estimate the size of PCR amplified fragments. Positive controls were Th*Lr9*, Olga, Tertsia (*Lr9*)*,* Th*Lr19*, Ulia (*Lr19*), Th*Lr24*, Scua (*Lr24*), KS90WGRC10 (*Lr41*), Pavon *Lr47* [14].

**Table 2:** DNA markers linked to effective leaf rust resistance genes in wheat.

Gene	Marker	Primer Sequence (5’-3’)	Fragment Size (bp)	Reference
*Lr9*	SCS5_550_	F: TGC GCC CTT CAA AGG AAG R: TGC GCC CTT CTG AAC TGT AT	550	[49]
*Lr19*	Gb	F: CAT CCT TGG GGA CCT C R: CCA GCT CGC ATA CAT CCA	130	[50]
*Lr24*	SCS73_719_	F: TCG TCC AGA TCA GAA TGT G R: CTC GTC GAT TAG CAG TGA G	719	[51]
*Lr41*	GDM35	F: CCT GCT CTG CCC TAG ATA CG R: ATG TGA ATG TGA TGC ATG CA	190	[31]
*Lr47*	PS10	F: GCT GAT GAC CCT GAC CGG T R: TCT TCA TGC CCG GTC GGG T	282	[52]

### 2.4. Adult Plant Resistance Screening

Adult plant resistance was studied in 2021 and 2022 in the field of Pushkin and Pavlovsk VIR Laboratories. Each sample grew in one row (length 1 m). Plants in the flag-leaf stage were sprayed with pathogen spore water suspensions and infected with the use of microchambers [39]. Samples highly susceptible to all four diseases under laboratory conditions were screened for adult resistance in the field only with use of microchambers method [39].

The types of reaction to *P. triticina* and *B. graminis* infections were scored on the 12th day after inoculation according to generally accepted scales [40,41] with modifications. Diseases with ratings of SNB and HLB were scored on the 7th day after inoculation according to above-presented scale and as described in [39].

## 3. Results 

### 3.1. Juvenile Resistance in Samples of Wild Wheat Species to the Diseases

According to the results of four independent experiments, the majority of test samples under study were susceptible to leaf rust, powdery mildew, Septoria nodorum blotch, and dark-brown leaf spot blotch at the seedling stage.

**Figure 1:**
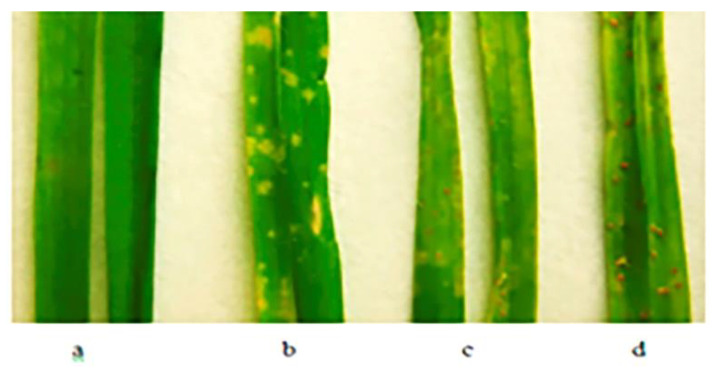
Seedlings of wild wheat species samples from the VIR collection after infection with population of *P. triticina*: (**a**) *T. boeoticum* k-66369; (**b**) *T. urartu* k-64777; (**c**) *T. araraticum* k-66372; and (**d**) *T. boeoticum* k-59178 (susceptible).

Only six entries of three species were highly resistant to the Northwest population of *Pt* (Table 3). Resistant *T. boeoticum* samples originated from Bulgaria and Turkey—*T. urartu* from Lebanon and *T. araraticum* from Turkey (Table 1). The reactions to leaf rust infection of three samples out these six, as well as the susceptible sample, are presented in Figure 1. 

Samples of *T. urartu* k-33870, k-33871, *T. boeoticum* k-27141, k-28283, k-58611, k-59159, k-59163, k-59178, *T. dicoccoides* k-15907, k-20403, and *T. araraticum* k-30258, k-30240, k-30234, k-31628 from the VIR collection have been previously described as resistant to *Pt* [35], as well as k-62492 of *T. boeoticum* [32] and were present in this study. In this work, only sample of *T. boeoticum* k-62492 was resistant at seedling stage to leaf rust.

Juvenile genes for resistance *Lr9, Lr19, Lr24, Lr41,* and *Lr47* are still effective in the Northwest of Russia [39] and were highly effective in this study. Leaf segments of six leaf rust resistant samples were inoculated with the single pathogen monopustule isolates virulent to wheat samples with five effective genes for resistance. All of these were highly resistant to these *Pt* isolates (Table 3), thus these samples evidently cannot have the single specific genes *Lr9, Lr19, Lr24, Lr41,* and *Lr47*.

**Figure 2:**
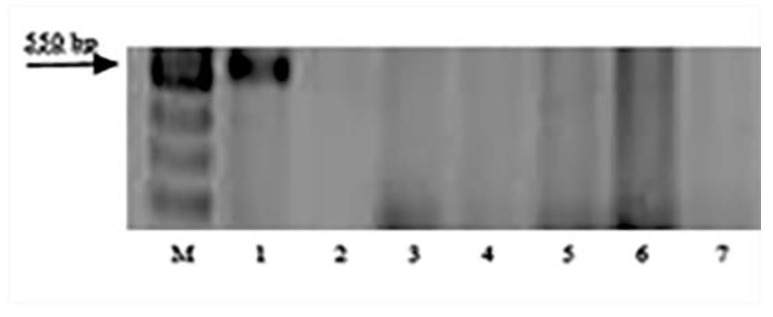
Amplification products of SCAR marker SCS5_550_ linked to leaf rust resistance gene *Lr9*: M—100 bp DNA Ladder; 1—Thatcher *Lr*9; 2—k-62492 *T. boeoticum*; 3—k-66369 *T. boeoticum*; 4—k-64777 *T. urartu*; 5—k-64782 *T. urartu*; 6—k-64783 *T. urartu*; and 7—k-66372 *T. araraticum*.

**Figure 3:**
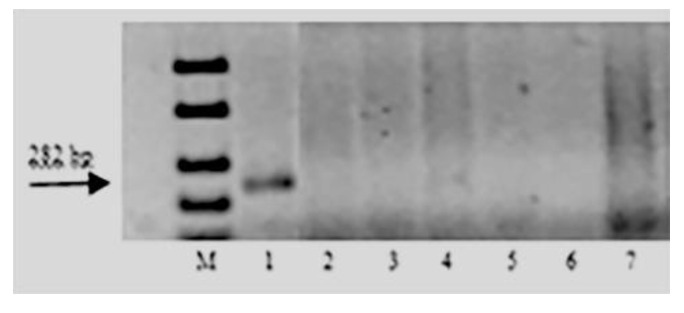
Amplification products of SCAR marker PS10 linked to leaf rust resistance gene *Lr47*: M–100 bp DNA Ladder; 1—Pavon *Lr47*; 2—k-62492 *T. boeoticum*; 3—k-66369 *T. boeoticum*; 4—k-64777 *T. urartu*; 5—k-64782 *T. urartu;* 6—k-64783 *T. urartu;* and 7—k-66372 *T. araraticum*.

The DNA markers specific to *Lr9*, *Lr19*, *Lr24*, *Lr41,* and *Lr47* genes were amplified only in wheat samples carrying the corresponding genes. None of the resistant to leaf rust sample of *T. boeoticum, T. urartu*, and *T. araraticum* had amplification products after PCR with primers to these genes’ markers. The electropherograms of the amplicons in samples with the SCS5_550_ marker primers and the PS10 marker primers are presented in Figure 2 and Figure 3.

Fifteen samples of *T. dicoccoides*, *T. boeoticum*, and *T. araraticum* were resistant to the population of *Bgt* sampled in the Northwest region of the Russian Federation (Table 4). All samples of *T. urartu*, including four that were earlier identified as resistant [35], were classified as susceptible to *Bgt* (type of reaction 3). Among 74 evaluated samples of *T. dicoccoides*, 11 were highly resistant to *Bgt*. These samples belonged to five botanical varieties and originated from Israel, Jordan, and Syria (Table 1 and Table 4). Resistant *T. boeoticum* samples were from Azerbaijan, Bulgaria, and Turkey; and the resistant *T. araraticum* entry was collected in Turkey.

Two samples of *T. urartu* (k-58504, k-33871), six samples of *T. boeoticum* (k-18424, k-28280, k-40117, k-58506, k-58489, k-59163), and five of *T. araraticum* (k-28132, k-28247, k-58668, k-30258, k-31628) present in this research were earlier described as resistant to powdery mildew at seedling stage [35]. Only one sample out of these—*T. boeoticum* k-58489, from Azerbaijan—was confirmed as resistant in this study.

**Figure 4:**
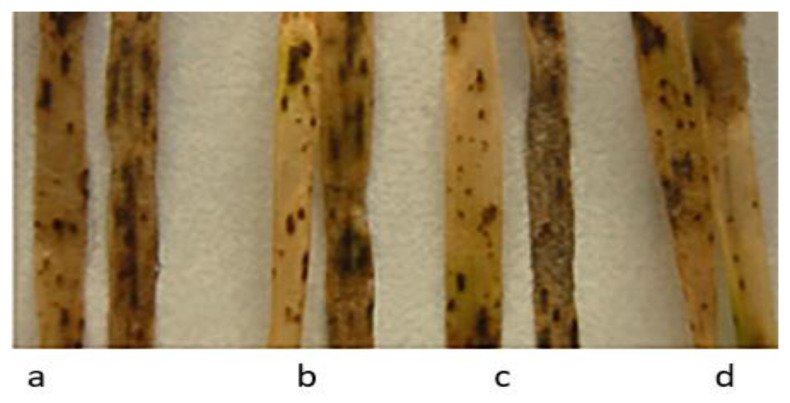
HLB development on seedlings of wild wheat species samples from the VIR collection after infection with a mixture of *B. sorokiniana* isolates: (**a**) *T. urartu* k-58504; (**b**) *T. araraticum* k-28244; (**c**) *T. boeoticum* k-62492; and (**d**) *T. dicoccoides* k-62364.

All studied samples of wild wheat species from the VIR collection, including 17 earlier described as highly or moderately resistant to dark-brown leaf spot blotch (*T. urartu* k-33871, k-58504, k-62458, k-62464, disease score 2 for all); *T. boeoticum* k-18424, k-40117, k-61660, k-62492 (disease score 1 for all); *T. dicoccoides*, k-61720, k-61828 (2), k-26117, k-62362, k-62364 (1–2); and *T. araraticum* k-28244 (2), k-30240 ), k-30268 (both 1–2), k-59940 (3) [36]), were susceptible to HLB in this work (disease ratings 5–6). The effects on disease seedlings of four samples in four wheat species from [36] are shown in Figure 4.

**Figure 5:**
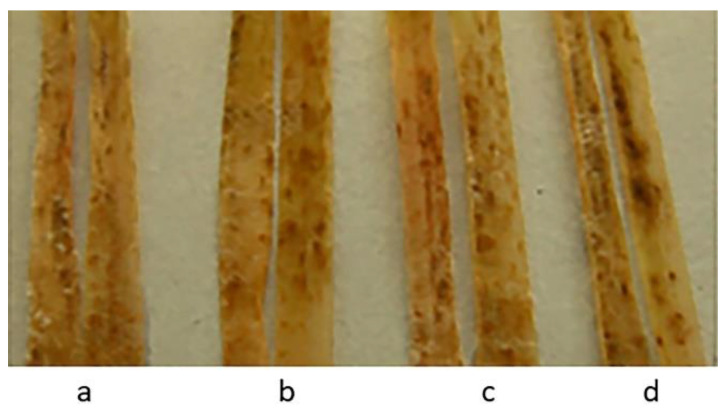
SNB development on seedlings of wild wheat species samples from the VIR collection after infection with a mixture of *S. nodorum* isolates: (**a**) *T. urartu* k-58497; (**b**) *T. urartu* k-66361; (**c**) *T. boeoticum* k-58506; and (**d**) *T. boeoticum* k-59178.

Similarly, all entries under study were highly susceptible to SNB (disease ratings 5–6). The disease development on four samples of *T. urartu* and *T. boeoticum*, the species most resistant to Septoria nodorum blotch according to [34] is shown in Figure 5.

### 3.2. Adult Resistance of Wild Wheat Species to the Diseases

Under field conditions, two samples of *T. boeoticum*, three of *T. urartu*, and one sample of *T. araraticum*, classified as resistant to the rust at seedling stage, displayed resistant types of reaction to the disease with two inoculation methods (Table 3). All other accessions were susceptible.

Fifteen samples of *T. dicoccoides*, *T. boeoticum*, and *T. araraticum* that had been classified as resistant to the Northwest population of *Bgt* under laboratory conditions, were shown to be highly resistant to the disease at the adult stages of plant growth (Table 4). Therefore, we did not identify accessions with only adult resistance to *Pt* or *Bgt*.

All samples were highly susceptible to SNB and HLB after inoculation of the microchambers in the fields of Pushkin and Pavlovsk laboratories of VIR with the respective pathogens.

## 4. Discussion

Leaf rust (*Pt*), powdery mildew (*Bgt*), dark-brown leaf spot blotch (HLB), and Septoria nodorum blotch (SNB) are economically significant diseases of bread wheat in many regions where the crop is cultivated. Leaf rust infections at even earlier stages may cause yield losses higher than 50% [4]. HLB was usually considered a harmful disease in non-traditional wheat-growing areas with high temperatures and humidity [53,54,55,56]. However, severe development of the disease has recently been reported in two regions of Russia [57,58]. HLB can result in yield losses up to 100% [6]. SNB is widespread across the world and losses from the disease can reach up to 50% [5]. *Bgt* is another common worldwide disease of wheat. Yield losses from powdery mildew under severe epiphytotic conditions can reach 50% or more [8]. For losses the maximum data are cited, indicating the possibility of lower or higher losses in the field. 

The economic value of these diseases has led to an intensive study of the bread wheat gene pool for resistance to them. The world collection of the N.I. Vavilov All-Russian Institute of Plant Genetic Resources is of great interest for the search of valuable breeding material, including wheat for resistance to harmful foliar diseases. Up until now, a large number of sources for resistance have been identified in the bread wheat collection of the VIR [34,36,59,60,61,62,63,64,65,66,67,68,69,70,71]. However, our earlier studies have shown that many VIR bread wheat samples previously identified in scientific literature as highly resistant are susceptible to *Pt*, SNB, and HLB [14,15,16,72,73]. The extremely limited number of genes is effective against *Bgt* in the Russian Federation [15,70,71], moreover samples earlier described as having very effective genes for resistance *Pm12*, *PmSp*, and *PmKu* were identified as susceptible to *B. graminis* populations from the Northwest region of the RF in 2022 (this study).

If the viewpoint on extremely narrow genetic diversity for effective resistance in *T. aestivum* to these diseases is correct, it is urgently important to use introgressive hybridization with closely related wheat species for its broadening. 

According to Vavilov’s concept of the centers of crops origin and diversity on utilization of plant genetic resources, the wild species of *Triticum* genus can be a very useful source of new effective genes for resistance to diseases for bread wheat breeding [12,13,33]. The Vavilov’ collection of this species has already been evaluated by several scientific groups for seedling resistance to *Pt* [14,15,16,32,35], HLB [14,32,36], SNB [14,32,34], and *Bgt* [35]. However, the data obtained on resistance to leaf rust, HLB, and SNB are very contradictory. So, among 56, 13, 28, 168 screened samples of *T. boeoticum*, *T. urartu*, *T. araraticum* and *T. dicoccoides,* 14%, 15%, 14%, and 13%, respectively, were resistant to leaf rust [35]. However, in the study of 258 samples of these species only four entries of *T. boeoticum* were found to be resistant to *Pt* [32]. For HLB resistance 17 entries of *T. boeoticum*, nine for *T. urartu*, eight for *T. araraticum* and 18 for *T. dicoccoides* were studied and eight, four, four, and five resistant samples were selected, respectively [36]. However, in our previous work no samples of those species with VIR catalogue number were resistant to HLB [32]. Yamaleev et al. [34] described *T. boeoticum* and *T. urartu* as the most resistant to *S. nodorum* species at the juvenile stage. Unfortunately, the authors did not provide detailed information on the characteristics of selected samples, which is why it is impossible to re-evaluate the resistance of particular entries. None of the samples of these species possessed any level of resistance in our previous study [32].

Here, 32 collection entries of *T. boeoticum*, 33 of *T. urartu*, 34 of *T. araraticum*, and 74 of *T. dicoccoides* were studied for effective seedling and adult resistance to four foliar diseases. Most samples under this study were susceptible to the diseases. Only six entries—two of *T. boeoticum* (k-62492, k-66369), three of *T. urartu* (k-64777, k-64782, k-64783), and one of *T. araraticum* (k-66372)—were highly resistant to *Pt* in seedling at the flag-leaf stages of plant growth. Notably, entry k-62492 was shown to be resistant at the seedling stage to *Pt* more than 15 year ago [32]. All samples of *T. dicoccoides* were classified as susceptible to the disease, although some genotypes of this species were described as resistant to leaf rust [74,75,76,77]; it is worth noting that resistant entries were often identified only after inoculation with extremely limited number of *Pt* isolates. Wheat line with gene *Lr64* from *T. dicoccoides* [77] was susceptible to the disease in this study. Resistance to the rust was earlier found in *T. araraticum* samples [78], though only one isolate of *Pt* was used for inoculation. Several wheat lines with genes for *Pt* resistance from this species were developed [79], but they were susceptible to at least two leaf rust races. After seedling inoculations with three *Pt* isolates many samples of *T. boeoticum* and *T. urartu* were found to be particularly useful for obtaining leaf rust resistance, moreover they were described as carriers of *Lr25* (gene from rye), *Lr28* (gene from *Aegilops speltoides* Tausch) and *Lr47* (from *Ae. speltoides*, too) [46]. *T. dicoccoides* k-17256 was identified as unaffected by the rust [37] but was found to be susceptible in this study. Samples of *T. urartu* k-33870, k-33871; *T. boeoticum* k-27141, k-28283, k-58611, k-59159, k-59163, k-59178; *T. dicoccoides* k-15907, k-20403; and *T. araraticum* k-30258, k-30240, k-30234, k-31628 from the VIR collection were identified as possessing resistance to leaf rust [35] but were found to be susceptible in this research. Several possible reasons could explain their susceptibility. We studied disease development on intact seedlings, and, during original work, leaf segments were infected with *Pt* on benzimidazole solution. Significant differences in types of reaction were often observed between these two methods of evaluation [14]. The changes in *Pt* population structure possibly could be the most important reason for the susceptibility of earlier resistant samples. From a practical viewpoint, it is evident that these entries cannot be recommended for breeding for resistance to leaf rust. The samples identified in this study can be used in interspecific hybridization to transfer the resistance genes to bread wheat. Genes *Lr9*, *Lr19*, *Lr24*, *Lr41*, and *Lr47* are still effective in the Northwest of the Russian Federation [39] (this study). The molecular markers for these genes were used to identify the corresponding genes in 6 samples of wild wheat species resistant to leaf rust. The DNA fragments specific to *Lr9* (550 bp) [49], *Lr19* (130 bp) [50], *Lr24* (719 bp) [51], *Lr41* (190 bp) [31], and *Lr47* (282 bp) [52] were amplified only in bread wheat lines and varieties carrying the corresponding genes. Phytopathological test with *Pt* isolates marked with virulence to the genes showed that resistance of *T. boeoticum*, *T. urartu*, and *T. araraticum* entries is controlled with new genes or possibly combinations of known effective genes for the trait.

Fifteen entries out of 97 studied were resistant at two ontogenesis stages to the population of *Bgt* sampled in the Northwest region of the Russian Federation in 2021–2022. They belong to three species (Table 3). All samples of *T. urartu,* including two earlier identified as resistant (k-58504, k-33871) [35], were classified as susceptible. Three samples (k-58489, k-62491, k-66370) of *T. boeoticum* from three countries were resistant to the disease, all others, including five—k-18424, k-28280, k-40117, k-58506, k-58489, and k-59163— shown to be resistant to *Bgt* according to Makarova et al. [35] have now been shown to be susceptible. Species *T. araraticum* was considered to be absolutely resistant (immune) or highly resistant to this disease [37]; this was confirmed for 28 accessions in 1993 [35]. The earlier susceptibility of most studied samples of this species has been already described [78]. In this study only one sample—k-64846 from Turkey—was found to be resistant, whereas seedlings and adult plants of others, including k-28132, k-28247, k-58668, k-30258, and k-31628, previously shown as resistant in [35], were severely affected by the modern *B. graminis* populations The main reason for the susceptibility of the earlier identified entries of three species is highly likely to lie in the drastic changes in *Bgt* population for virulence during the last 18 years; confirmation of this could be seen in high frequencies of *Bgt* isolates virulent to the previously highly effective genes *Pm12*, *PmSp*, and *PmKu* [80]. In this study, the majority of entries resistant to the *B. graminis* population belong to *T. dicoccoides* and originated from Israel (nine samples), while two samples were entered into the collection from Iordania and Syria. In an earlier study of wild wheat species from samples other than VIR’s genetic collections, the resistance to *Bgt* was found, for instance, in *T. araraticum* [78], *T. boeoticum*, *T. urartu* [46], and *T. dicoccoides* [81,82], but only a limited number of fungal isolates (1–3) were used for inoculations. The samples identified in the present work as highly resistant to complex powdery mildew populations, especially those of the *T. dicoccoides* species, are of great interest for hybridization with bread wheat. Preliminary study of the resistance genetic control and possible allelic relationships of genes for the trait is necessary to prevent transfer of identical genes in *T. aestivum* genotypes. Notably, the effective resistance of identified accessions could not be controlled by 23 single genes listed in the Materials and Methods section because they are not effective against modern populations of *Bgt* from the Northwest region of the RF.

Earlier, samples of *T. urartu* k-33871, k-58504, k-62458, k-62464; *T. boeoticum* k-18424, k-40117, k-61660, k-62492; *T. dicoccoides* k-26117, k-61720, k-61828, k-62362, k-62364; and *T. araraticum* k-28244, k-30240, k-30268, k-59940 have all been described as resistant (eight accessions) or moderately resistant (nine accessions) to HLB [36]. All these samples were susceptible to the disease in the present work. At least three possible reasons could explain this serious inconsistency of the results. To identify the resistance, we inoculated intact seedlings with the pathogen while Mikhailova et al. [36] studied resistance of leaf segments on benzimidazole. Then we scored the ratings on the seventh day after inoculation vs. the third day after infection with *B. sorokiniana* conidia in [36]. Finally, we inoculated leaves with a mixture of five pathogen isolates, only one was used in Mikhailova et al. [36] and then possibly of lower aggressiveness. None of the samples from newer entries of the collection was resistant to the disease in seedlings and at adult stage. Hence, the genetic diversity for HLB resistance in wild wheat species is extremely low, as has been proposed earlier [14,15,16,32,72].

In this study we also determined that all samples were highly susceptible to SNB (disease ratings 5–6) under the laboratory and field conditions. Yamaleev et al. [34] studied seedling resistance in *Triticum* species and identified wild wheat species *T. boeoticum* and *T. urartu* as the most resistant to SNB. Unfortunately, information on the number of studied samples in each species and variability for SNB resistance among samples was not presented in the publication [34]. As mentioned above, no one entry of those species possessed any level of seedling resistance to *S. nodorum* in our study. The sources of SNB resistance were also found also in some genetic collections of wild wheat species, namely among samples of *T. dicoccoides* [83,84]; however, in the present work all 83 entries were shown to be highly susceptible. Similar to HLB, our current research confirmed conclusions of a very narrow genetic diversity for effective seedling and adult resistance to Septoria nodorum blotch in wild wheats from the VIR collection [14,15,16,32].

As a result, we selected entries of wild *Triticum* species from the world VIR collection with effective resistance to populations of leaf rust in three species and to powdery mildew also in three species. These samples are of great interest for interspecific hybridization with bread wheat in order to develop new valuable donors for resistance to two harmful foliar fungal diseases. Additionally, we here confirm the extremely narrow genetic diversity for resistance to SNB and HLB in wild wheats from the Vavilov’ collection [14,15,16,32]. Hence, our data confirm the basis position of Vavilov’s concept of the wheat’s diversity for resistance to diseases [12,13,33] at least to those caused by biotrophic pathogens, and not caused by hemibiotrophic pathogens. It should be especially mentioned that all Vavilov’s concepts concerning the genetic diversity of wheats for disease resistance was based on experimental data on resistance to obligate parasites.

Besides practical conclusions, our data could be regarded in the view of another outstanding concept from Vavilov— the “keys for finding plant immune forms” or laws of natural immunity (resistance in our modern terms) [33,85,86,87,88]. This concept is the theoretical and practical basis for the identification of cultivated plants and their relatives’ sources and donors of effective resistance to pests and diseases. Although even Vavilov outlined that these laws are not absolute [33] and later some contradictions were found with regards to the current data on plant resistance [89]. This aspect of his conclusions is supposed to be absolutely actual to this day. The general six points of this concept’s last version [33,87] are briefly discussed below in context of our experimental data.

First to begin the breeding for resistance to diseases, it is necessary to know the biology of the parasite and, above all, its specialization. The narrower the specialization of the parasite in limits of plant genera and species, the greater the chances to identify resistant forms within individual species [33,88]. For example, in the works of Vavilov, many bread wheat varieties resistant to a highly specialized pathogen of yellow rust have been identified, and, conversely, the vast majority of varieties were susceptible to stem rust, the causal agent of which has a wide range of hosts. At the same time, a relatively large number of examples can be presented that refute this conclusion for cases of intraspecific diversity for effective resistance in widely cultivated species today. For instance, in the VIR collection there are currently no barley samples that are resistant (at least in the juvenile stage) to a highly specialized pathogen of leaf rust, but there are several accessions with a fairly high level of resistance to dark-brown leaf spot blotch (a pathogen with extremely wide specialization) [14,90]. In the VIR collection there are many samples of bread wheat that are resistant to leaf rust, but they are all created based on the use of introgressive hybridization [14,16]; if one were to consider only those genotypes without alien genes, then the number of varieties resistant to the rust (narrow specialized parasite) would not be more than those resistant to dark-brown leaf spot blotch. It should be especially noted that, if replacing the term “within species” with the term “between species”, or even further with “between genera”, there is no doubt about the universality of the law. Furthermore, when discussing genetic diversity for effective resistance to disease in plant species that have never been grown widely, the correctness of this aspect of Vavilov’s natural immunity (now resistance) concept is absolutely evident. Therefore, in this study of resistance to diseases in four wild *Triticum* species (173 accessions) we identified six samples of three species resistant to wheat leaf rust (narrow specialized pathogen) and 15 entries resistant to powdery mildew (also a narrowly specialized pathogen), but all of these were highly susceptible to dark-brown leaf spot blotch and septoriosis (both of which are caused by parasites with low specialization).

The second point of the law claims that the most highly contrasting differences in resistance are revealed in plants that are cytogenetically sharply differentiated into different species [33,88]. As one of the examples, Vavilov regarded the exceptional resistance of einkorn wheat to leaf rust and resistance to crown rust and smut in genetically separate species of wild and cultivated oats [88]. This point of the concept is partially supported by our present data. All bread and durum wheat from the VIR collection were susceptible to *Pt* and *Bgt* (not considering samples with alien genes for the resistance) [14,15]; however, six and 15 samples of wild species cytogenetically differing from *T. aestivum* and *T. durum* Desf. were highly resistant to these diseases at the two stages of plant growth, respectively, in this study. However, in terms of reactions to weakly specialized pathogens (*B. sorokiniana* and *P. nodorum*) we did not find differences in the frequencies of resistant forms among many *Triticum* species [14,15,16] including those studied for this research.

The most highly contrasting differences in resistance are detected in the most highly contrasting environmental conditions; there is a certain relationship between ecological differentiation of species and differences in the response of varieties to various parasitic diseases [88]. Therefore, according to Vavilov, most varieties and ecological–geographical groups of barley are very susceptible to barley leaf rust; however, a large number of immune forms have been identified among the material from the Mediterranean region [33,88]. Similarly, resistance to yellow rust, powdery mildew and other diseases was found in the Abyssinian subspecies of barley. Vavilov also noted that the accessions of bread wheat from Central and Southern China regions, but not from Western China, included a number of forms with drastically pronounced resistance to leaf and yellow rusts [88]. Vavilov explained this fact by creating resistant forms under conditions of humid and warm climate, favorable for the development of these diseases. Without questioning the correctness of this law’s point at the time of its development, it should be noted that all VIR wheat samples from China are susceptible to modern populations of the leaf rust pathogen from the RF [14]. Additionally, barley samples (including local forms from different regions) lack high level of resistance to leaf rust [14,90]. One of the most plausible explanations of this contradiction is a change in virulence structures of phytopathogen populations because of breeding achievements in the creation of resistant varieties, as well as changes in the virulence of pathogens under the effects of global climate change. The data obtained in our work are difficult to consider regarding this point of Vavilov’s concept because of the relatively small number of identified resistant samples. However, it is necessary to emphasize that accessions of *T. boeoticum* resistant to *Pt* originated from two different regions, resistance to *Bgt* from three regions (Table 1) and samples of *T. dicoccoides* resistant to *Bgt* were also from three different regions (Table 1). Vavilov [33] described samples of *T. dicoccoides* from Syria as resistant to leaf rust and that those from Palestine could be differentiated for their resistance; however, in this study all accessions of this species were susceptible. Thus, we were not able to find any close relationship between resistance of samples and their origin possibly due to limited quantity of plant material from certain regions.

The next point declared that group, or complex, immunity (i.e., resistance, in view of modern terminology) is a quite real fact that is widespread in nature and can therefore be used in practical breeding [33,88]. Species resistant to a single disease, are often resistant to many other diseases. Therefore, *T. timopheevii* is highly resistant both to leaf and yellow rusts, loose smut, powdery mildew, frit, and Hessian flies [88]. Complex resistance is also characteristic for *T. monococcum* [33]. There should be no doubt that, for this point of Vavilov’s concept, it is necessary to underline the key phrase “resistant species” i.e., species for which all accessions are resistant to certain diseases. When there is intraspecific differentiation for resistance this point of law of natural immunity should be considered as doubtful. For instance, in this study of 173 accessions of *Triticum* species, we found six samples resistant to *Pt* and 15 to *Bgt*, but none were resistant to more than one disease. At the same time resistance to several diseases is not completely rare. For instance, bread wheat genotypes, created as a result of interspecific hybridization by transferring into their genomes relatively large segments of alien chromosomes, are often resistant to several diseases. We have also left behind the discussion of resistance (immunity) to parasites that are non-pathogenic to a given host species (in this case any sample possesses complex immunity to thousands of diseases).

Distribution of immune and susceptible species and varieties is not an accident. Knowing the evolution of a certain cultivated plant, its differentiation into certain genetic and ecological–geographical groups, it is possible to foresee to a large extent the location of immune forms interesting for breeding [33,88]. This conclusion, as Vavilov indicated himself [88], is a logical consequence from the first four concept points, so its correctness does not require additional proof beyond the consideration of our remarks given above (the first four statements).

Additionally, as a final point of Vavilov’s law we will notice only his brilliant foresight. That, in the near future, we will be witnesses to changes in our varieties, in the sense of radical increases in their immunity (resistance), by means of distant hybridization [33,88]. Indeed, in many cases, there have been successes in the modern breeding of cultivated plants for effective resistance to fungal diseases (including complex diseases), the causal agents of which are able to spread over considerable distances (and therefore, theoretically can overcome existing resistance due to the mutational process and selection) and are associated with the success of introgressive hybridization. The samples of wild *Triticum* species identified in this study as resistant to *Pt* and *Bgt* could serve as very useful initial material in this direction of wheat breeding.

## Figures and Tables

**Table 1:** Characteristics of wild wheat species samples studied for resistance to the diseases.

Species	2n	Genome	Country	VIR Catalogue No, kk-
*T. urartu* Thum. ex Gandil.	14	А^u^	Armenia Iran Jordan Lebanon Syria Turkey	33870, 33871 58497 66361, 66362 64772–64780, 64782–64784 62458, 62464, 62479, 65752 58504, 64768–64771, 64786–64788, 64790, 66371, 67176, 67390
*T. boeoticum* Boiss.	14	А^b^	Armenia Azerbaijan Bulgaria Greece Iran Iraq Syria Turkey Russia Ukraine	58506, 58509, 58511, 58674, 59166, 64831, 64832, 65530, 65531, 65538, 65540 28280, 28283, 58489, 59159, 59163 62491, 62492 61660 65753 40117, 65543 65754, 65755 27141, 65952, 65970, 66369, 66370 18424 59178, 65535
*T. dicoccoides* (Koern. ex Aschers. et Graebn.) Schweinf.	28	А^u^B	Iraq Israel Jordan Lebanon Turkey Syria	42632, 67163 5199, 15903, 15907, 17256, 17259, 20403, 26117, 26119, 42633, 61678, 61681, 61690, 61692, 61697, 61707, 61709, 61720, 61722, 61723, 61730, 61732, 61742, 61750, 61753, 61754, 61761, 61763, 61764, 61766–61768, 61778, 61779, 61781, 61782, 61785, 61794, 61805, 61811, 61812, 61818, 61820, 61828, 61832, 61833, 62329, 62333, 62338, 62345, 62347, 67164, 67177 65748, 65550–65552, 65750, 67174, 67175 65751 66434, 66436 17157, 61712, 61714, 61717, 61719, 62362, 62364, 62366, 67161, 67162
*T. araraticum* Jakubz.	28	А^b^G	Armenia Azerbaijan Iraq Turkey	28132, 30258, 31628, 59940, 61654, 64838, 65541, 65542, 66115 28244, 28247, 30234, 30240, 30268, 58668 40120, 64836, 64837, 64847–64850, 66103–66106, 66108, 66110 66111–66114 64846, 66372

**Table 3:** Samples of wild wheat species from the VIR collection that are highly resistant to leaf rust.

VIR Catalogue No. kk-	Species	Botanical Variety	Growth Stage							
			Seedlings, Inoculated with Population Clones Virulent to Genes *						Flag Leaves, Inoculated with	
				*Lr9*	*Lr19*	*Lr24*	*Lr41*	*Lr47*	Microchambers	Spraying
62492	*T. boeoticum*	*pseudoboeoticum*	0;−1	0	0	0	0	0	0;	0;
66369	*T. boeoticum*	*pseudoboeoticum*	0	0	0	0	0	0	0	0
64777	*T. urartu*	*spontaneoalbum*	0;−1	0	0	0	0	0	0;	0;
64782	*T. urartu*	*albonigricans*	0;−1	0	0	0	0	0	0;	0;
64783	*T. urartu*	*spontaneoalbum*	0;−1	0	0	0	0	0	0;	0;
66372	*T. araraticum*	*kurdistanicum*	0–2	0	0	0	0	0	0–2	0–2

* Five independent clones for each gene.

**Table 4:** Samples of wild wheat species from the VIR collection that are highly resistant to powdery mildew.

VIR Catalogue No, kk-	Species	Botanical Variety	Growth Stage		
			Seedlings	Flag Leaves, Inoculation with	
				Microchambers	Spraying
61712	*T. dicoccoides*	*dicoccoides*	0	0	0
61720	*T. dicoccoides*	*vavilovii*	0	0	0
61732	*T. dicoccoides*	*aaronsohnii*	0	0	0
61753	*T. dicoccoides*	*namuricum*	0	0	0
61764	*T. dicoccoides*	*vavilovii*	0	0	0
61767	*T. dicoccoides*	*vavilovii*	0	0	0
61768	*T. dicoccoides*	*arabicum*	0	0	0
61805	*T. dicoccoides*	*namuricum*	0	0	0
61811	*T. dicoccoides*	*aaronsohnii*	0	0	0
61833	*T. dicoccoides*	*arabicum*	0	0	0
67175	*T. dicoccoides*	*dicoccoides*	0	0	0
58489	*T. boeoticum*	*pseudoboeoticum*	0	0	0
62491	*T. boeoticum*	*kovarskii*	0	0	0
66370	*T. boeoticum*	*boeoticum*	0	0	0
64846	*T. araraticum*	*araxicum*	0	0	0

## Data Availability

Not applicable.
